# Exploring the strategies that midwives in British Columbia use to promote normal birth

**DOI:** 10.1186/s12884-017-1323-7

**Published:** 2017-06-05

**Authors:** Michelle M. Butler

**Affiliations:** 1 Department of Family Practice | Midwifery Program, University of British Columbia, 320 – 5950 University Boulevard, Vancouver, BC V6T1Z3 Canada; 2 Faculty of Science and Health, Dublin City University, Glasnevin, Dublin 9 Ireland

**Keywords:** Midwifery, Normal birth, Interventions, Caesarean section, British Columbia

## Abstract

**Background:**

Rates of normal birth have been declining steadily over the past 20 years, despite the evidence of the benefits to mother and baby. This is most obvious in steadily increasing caesarean section rates across countries and studies of the factors involved suggest it may be more to do with the organization of maternity care and the preferences of healthcare providers than changes in maternal or demographic conditions. The proportion of women in British Columbia (BC) receiving care from a midwife continues to grow and there is a particular focus on promoting and supporting normal pregnancy and birth in the midwifery philosophy of care. In BC, women receiving care from a midwife are less likely to have a caesarean section and other birth interventions.

**Methods:**

An interpretive approach, based on interpretive phenomenology was used to explore the experiences of midwives in BC of normal birth and the strategies that they use to keep birth normal. Fourteen experienced midwives were purposively selected from across the range of practice, geographical, and rural/urban contexts to participate in depth interviews. Data were analyzed using Thematic Network Analysis.

**Results:**

Seven key themes were identified in the data: working with women from the early pregnancy, informing choice, the birth environment, careful watching and waiting, managing early labour, helping the woman to cope with labour, and tools in the tool kit.

**Conclusions:**

Midwives in BC work closely with women from early pregnancy to prepare them for a normal birth, and as “instruments of care” they adopt a range of approaches to support women to achieve this. The emphasis on continuity of care in the BC model of midwifery care plays a vital role in this.

**Electronic supplementary material:**

The online version of this article (doi:10.1186/s12884-017-1323-7) contains supplementary material, which is available to authorized users.

## Background

**Fig. 1:**
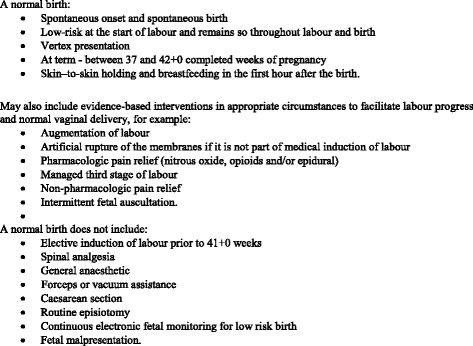
Definition of normal birth in Canada

**Fig. 2:**
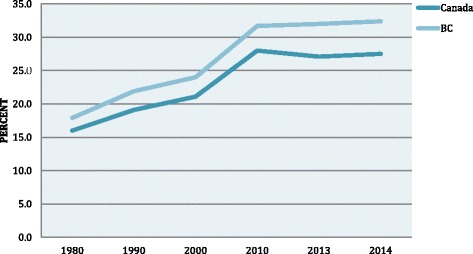
Caesarean Births in Canada and BC, 1980–2014. *Source*: *CIHI* [4], *PSBC* [5], *Statistics Canada* [6]

In Canada, the World Health Organization’s definition of normal birth (see Fig. 1) has been accepted and extended to also include the opportunity for skin–to-skin holding and breastfeeding in the first hour after the birth, and evidence-based interventions in appropriate circumstances to facilitate labor progress and normal vaginal delivery [1]. Although there is concern about the unnecessary use of all interventions in labor, the major debate in Canada, as elsewhere, is on Caesarean section (CS). CS is seen to be at the pinnacle of interventions, often resulting from earlier technical interventions that have had unintended effects, that subsequently need to be addressed with further interventions. This is often referred to as the “cascade of interventions” [2]. Currently 1 in 5 women in the world give birth by CS [3]. CS rates in 2014 were 27.5% in Canada [4] and 32.4% in BC [5] and have risen steadily from 16.0% (Canada) and 17.9% (BC) in 1980 [6] (see Fig. 2).

While CS will be essential in some cases to achieve a safe outcome, the real concern is about the growing rate of unnecessary CSs and its impact on women and their infants. For example, CS is associated with higher rates of maternal mortality, morbidity, infection, hemorrhage, thrombosis, and complications in subsequent pregnancies. For the infant, it is associated with increased mortality and morbidity, admission to neonatal unit, respiratory distress syndrome, and difficulty bonding and breastfeeding [7]. More recently, studies have raised concern on the association of CS with longer-term chronic conditions such as asthma, diabetes, cancer and obesity and on stem cell epigenetics [8, 9]. It is estimated that an appropriate CS rate is between 10% and 15%; rates above 15% “may result in more harm than good” [10]. From a health systems’ perspective, there is also concern about the costs that unnecessary CSs impose on financially stretched health systems [3].

A taskforce established in British Columbia (BC) in 2006 to investigate the increasing rates of CS concluded that although the factors that are often blamed on increases in CS rates such as older maternal age, hypertension, diabetes, obesity and multiple pregnancy had increased, “the rate of caesarean birth is rising faster than medical or demographic conditions would justify” [11]. In the UK, Marshall et al. [7] had similar findings and concluded that “the most likely reason for variation in rates is difference in thresholds for intervention and variations in preferred models of care at institutional and practitioner levels” (p.333). A systematic review of strategies to reduce the rate of Caesarean birth in low-risk women [12] concluded that no single strategy was uniformly successful in reducing CS but the evidence was weak—suggesting more research is needed to begin to identify effective strategies to reduce CS. A systematic review of non-clinical strategies to reduce CS [13] also highlighted the limited research evidence available.

Midwifery was established as a regulated profession in BC in 1998 and demand for care from a midwife continues to grow—in 2015, 21% of women had care with a midwife [14]. Midwives in BC work as autonomous primary care providers and the midwifery model of care emphasizes continuity of care and informed choice, including choice of place of birth. The midwifery philosophy emphasizes pregnancy and birth as normal and profound life experiences and the role of the midwife in keeping birth normal [15, 16]. Studies conducted in BC have shown that women who receive care from a midwife at home or in hospital, are less likely to have procedures during labour such as caesarean section, narcotics analgesia, electronic fetal monitoring, amniotomy, and episiotomy, compared with women who give birth in hospital attended by a physician [17–19].

Continuity of care is a key and deliberate feature of the model of midwifery care in BC and to achieve this, midwives work as solo practitioners or in teams of up to four midwives, each midwife can provide care for a caseload of up to 60 women each year, and each midwife is compensated per “course of care” through the province’s universal health insurance (Medical Services Plan). Each woman will see her midwife several times before the birth at visits that last for about 45 min. Most women who have care with a midwife will know the midwife who attends them for the birth and women have the choice of place of birth—hospital, home, or another appropriate setting. Women receiving midwifery-involved care (a midwife was involved in their care but may not have been the delivery provider) in BC in 2014/15 were more likely to have a vaginal birth (79.7% versus 67.6% for all care providers) and less likely to have a caesarean section (20.3% versus 32.4% for all care providers) [14, 20].

This study set out to examine the experiences of midwives in BC of normal birth, the challenges that they experience to keeping birth normal and the strategies that they themselves use in their practice.

## Methods

An interpretive approach based on interpretive phenomenology and involving in-depth interviews, was used to explore midwives’ experiences of promoting and supporting normal birth (see Additional file 1 for the interview guide). This approach enables the researcher to create a “dialogue between practical concerns and lived experience through engaged reasoning and imaginative dwelling in the immediacy of the participants’ worlds” [21] (p.99).

### Sample

On the basis that the context of practice in BC would be important in relation to the challenges that midwives would experience and the strategies that they would use, midwives with at least 5 years’ experience as a midwife in BC were selected from a list of registered midwives, to reflect possible variations in experiences between midwives working in urban and rural locations across BC (lower mainland, Vancouver Island, the interior and Northern BC), and between those working as solo, midwifery group and collaborative practitioners (midwives and family doctors sharing a caseload). Following approval from the university Behavioural Research Ethics Board, 16 midwives were approached, provided with verbal and written information on the study and 14 consented to participate.

### Data collection

Interviews provide “a unique access to the lived world of the subjects, who in their own words describe their activities, experiences and opinions” [22] (p.10). Individual semi-structured interviews were used for this reason and to enable the researcher to further explore and seek clarification around themes raised by participants. The majority of interviews took place face-to-face in midwives’ place of work or home, two interviews were conducted on Skype^TM^ and two were conducted by telephone. Interviews lasted for between 45 min and an hour and were recorded digitally. Recordings were transcribed for analysis. Interviews began by exploring what midwives understood by the term normal birth, participants’ experience of normal birth in their daily practice as midwives, any particular challenges they experience and the strategies that they use to maintain normal birth. The focus of this paper is on the strategies that midwives used to maintain normal birth.

### Analysis

Thematic network analysis [23] was used to reduce and explore the text, and to integrate the themes identified in this exploration. The process began by the researcher reading transcripts to become very familiar with the content and identify patterns in the data. On subsequent readings codes were applied to the data to reflect the themes emerging (basic themes). Similar themes were grouped together to form categories (organizing themes) and these were further clustered to form *global themes*. In this way, the data were explored, thematic networks were constructed and explored, and patterns were summarized and interpreted.

### Reflexivity

Berger [24] describes reflexivity as a “process of a continual internal dialogue and critical self-evaluation of [the] researcher’s positionality as well as active acknowledgement and explicit recognition that this position may affect the research process and outcome” (p.220). There will be several potential sources of bias in qualitative research, given the close connection between the researcher and participants and the use of the human as instrument [25]. These include power imbalances, and perceptions and misconceptions about roles and interests in the topic being researched. In this study, the researcher was a midwife but had not practised in BC and occupied a leadership position in academia. To minimize any biases that could arise, the research process involved both prospective (thinking about how the researcher influenced the participants) and retrospective reflexivity (thinking about how the participants influenced the researcher) [26]. From the prospective perspective, participants were informed in invitation letters of the purpose of the research, why they had been selected to participate, that they were under no obligation to participate in the research, and were assured of their anonymity. Before beginning interviews, participants were again assured of their anonymity and encouraged to be open about their experiences. From the retrospective perspective, an open questioning style was used in interviews and ideas and issues raised by the participants were returned to them for further probing. The researcher was careful to seek clarifications about assumptions made and to keep participants’ views separate from her own opinions and presuppositions. Interview transcripts were analyzed using software (NVivo, Version 10.2.1, QSR International Pty Ltd.) to allow all data to be dealt with systematically, to keep an accurate record of the themes identified, and to keep memos of observations that emerged in the course of analysis.

## Results

**Fig. 3:**
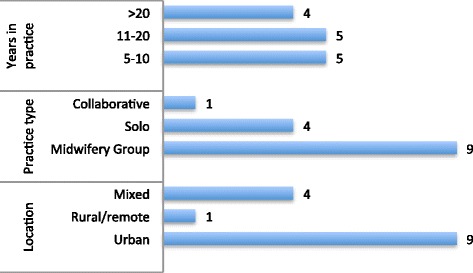
Participants (n)

The 14 midwives who participated in the study were from across the range of practice types, urban, rural and remote contexts, and geographical locations in BC and nine participants had more than 10 years experience as midwives (see Fig. 3).

Interviews began by exploring midwives understandings of the term *normal birth*. Eleven of the fourteen midwives interviewed raised concerns with the term *normal*, suggesting the term was academic, only classified after the fact, or culturally defined. Eight midwives preferred the term *physiologic*, and seven midwives referred to normal birth as an *outcome* and spoke about working towards a normal vaginal birth.

Two midwives described how what they considered to be normal shifted over time:

*To be honest I feel like I had a really clear definition at one point. It was really clear to me that normal birth was no intervention*, *healthy woman*, *healthy baby* … *I feel like my definitions are shifting quite significantly in terms of what is a normal length of birth*, *what is the normal length of pushing* … *why if everything is going well and baby and mum*, *mum and baby are doing fine*, *then can we stretch those meanings of normal a little bit more*?

Six midwives spoke about the importance of experience and confidence in their ability to support a normal birth. They talked about how, over time, they became more confident to work to keep the birth normal and to question procedures and protocols that *work against normal labour*. They also spoke about the benefits of their experience in terms of developing a range of skills to deal with different situations and in terms of being more tuned into what is happening in particular scenarios.

### Strategies used by midwives to keep birth normal

**Fig. 4:**
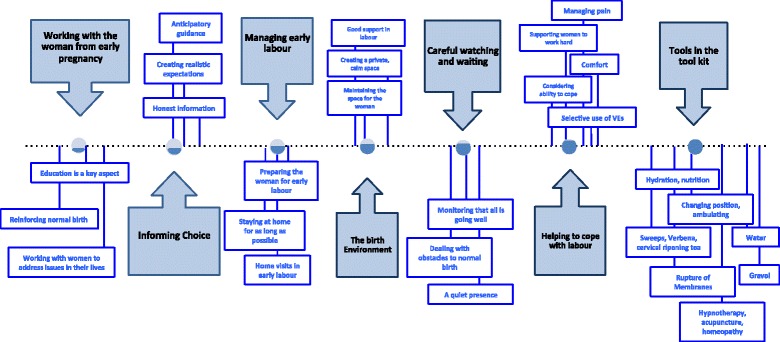
Strategies that midwives use to keep birth normal along the care timeline

Participants identified and discussed the particular strategies that they used to keep birth normal. Seven key organising themes were identified relating to: 1. Working with women from the early pregnancy, 2. Informing choice, 3. Managing early labour, 4. The birth environment, 5. Careful watching and waiting, 6. Helping the woman to cope with labour, and 7. Tools in the tool kit (see Fig. 4).

#### Working with women from the early pregnancy

Eleven midwives talked about working with the woman from early pregnancy towards a normal birth, for example:

*I have this expectation for every delivery that I go to that it will be normal in the end. There may be little challenges here and there but that*’*s what my experience and training is for*, *to overcome those challenges and still allow women to have a vaginal delivery. Of course there are some cases that are different*, *you know if there is major pathology then of course*, *I have to let go of that expectation but throughout*, *or let*’*s say from the first day that I see a woman that*’*s what I work towards. I have 9 months so it*’*s quite a bit of time to prepare her and myself getting to know her*, *have a good delivery with you know a vaginal birth in the end and no major complications*.

A key theme in all of this was on-going education:

*We do a lot of prenatal education*, *we talk a lot about what labour looks like*, *how to prepare for it*, *we talk to the partners about how to support them in labour*, *we talk about all the different kind of management techniques and comfort measures and we talk to them about pain relief options all the way from non*-*pharmaceutical to pharmaceutical and epidurals and what*’*s available* … *so I really prepare them for the idea that we*’*re going to do a lot of this outside the hospital even if they*’*re planning a hospital delivery that they have the tools and the resources that they need to cope with labour*, *that labour*’*s a very normal thing and uh*, *we make sure that we*’*re available to them*.

One midwife referred to *anticipatory guidance*, where midwives provide women with timely and clear information, so that they know what to expect and become confident in how they will deal with different scenarios. Another midwife referred to changing women’s perceptions over time and on-going reinforcement of birth as a normal event. Three midwives highlighted the need to work with women to create realistic expectations. Participants referred to working with the woman to address problems in their lives that are likely to impact on the birth and focusing on the woman having a healthy pregnancy so that they would be physically fit for labour and birth. It was also suggested that the longer prenatal appointments that women have with midwives is helpful in being able to provide this information.

#### Informing choice

Midwives spoke about the challenge involved in providing balanced information to women so that they can make an informed choice, while also supporting normal birth. They spoke about presenting the evidence and guidelines, and supporting women to make informed choices. They suggested it is easy to be *hands*-*off* when everything is going to plan but there is a particular challenge in informing choice when problems arise for women who do not want interventions. It was suggested that women should be supported to have appropriate interventions in labour, if they will benefit the woman:

[*If*] *she*’*s communicating that she*’*s really really tired*, *I have this you know gut feeling that if I rupture her membranes things are going to move really fast and there*’*s going to be a baby and she can just snuggle her baby and go to sleep then that may be an intervention I use in that moment*, *as something I might offer her and explain it that way*, “*it*’*s an intervention*, *it can help things move along quicker*, *is that something you want to try*?”. *These are the risks and benefits*.

One midwife highlighted the importance of *being authentic* and women having a choice even if it is not to have a normal birth, for example a woman wanting an elective caesarean section. Three participants referred to providing honest information so that a woman can make an informed choice around induction:

*I*’*ve had people say things like when you get to 42 weeks your risk of stillbirth will double and then just leave it at that*, *whereas we*’*re very conscious to say well yes your risk of stillbirth will double but this is what it is doubling from and to and these are the actual stats*, *these may be some other factors to bear in mind when you*’*re making this decision. So I suppose it*’*s about using the information accurately and not using it in a frightening way or a way to be able to coerce women into what would be the community standard. So you know if I just stop at your risk of stillbirth doubles at 42 weeks*, *what sane woman wouldn*’*t choose to be induced but if we then go onto say what that actually involves then I would find*, *we have a very low induction rate compared to some places*.

One participant highlighted the importance of balancing the aim of achieving a normal birth with other priorities that women have, for example when providing care for a woman with a history of trauma or intimate partner violence.

#### Managing early labour

Participants spoke about the importance of managing early labour well, so that women are in a good position to go into active labour. They spoke about preparing women for what to expect in early labour and how to prepare for it:

… *we talk a lot about what labour looks like*, *how to prepare for it*, *we talk to the partners about how to support them in labour*, … *especially for the primips* … *that prodromal labour stuff I mean that*’*s a mental game and that*’*s why a lot of women end up in the hospital too early*, *so we do*, *we do a lot of home support for that and phone calls and making sure they*’*re well prepared for that and coping with that* … *when I*’*m talking with them prenatally about what early labour looks like* versus *active labour*.

Midwives also talked about how they support the woman to get some rest and to eat and drink in early labour so that they are ready for active labour:

… *I really emphasize very strongly with my clients about how to manage their early labours and sleep in early labour and I encourage them to consider Gravol* [*Dimenhydrinate*] … *for sleep*, *even like I said certainly the first night if they*’*re starting early labour at night and even sometimes the next day like late in the day or early in the evening*, *if they*’*re taking their time. I just find that rest can have a huge effect in beneficial ways and also making sure that they eat and drink and making sure that they*, *hoping and helping them to ignore as much as early labour as they can*.

Encouraging women to stay at home for as long as possible was a particular strategy used to keep birth normal, and to reduce the likelihood of interventions:

*Well I think there*’*s research to show that the earlier you go into hospital the more likely you*’*ll end up with intervention cascade so one of the things I try to do*, *because we*’*re watching more and starting to document more*, *if they*’*re in a big hospital system*, *then I think there*’*s more of a tendency and I think midwives are no different*, *as a practitioner you kind of think you have to be doing something whereas at home you can go listen to baby*, *do the blood pressure*, *listen you know and reassure and make sure she*’*s eating and drinking but it*’*s not so much okay now we*’*re going to do a check again in 2 or 3 or 4 h for a vaginal exam and see if there*’*s any progress when you*’*re starting at*, *you*’*re not really in labour. So let labour establish itself before you start thinking there should be progress* …

Five midwives talked about the value of visiting women at home in early labour to assess, reassure and support them.

#### The birth environment

Participants spoke about the importance of the birth environment to supporting normal labour and birth. Midwives suggested that the home was the place that was most conducive to having a normal birth and in some hospitals birthing rooms were available with pools and a low-tech environment. However, participants also talked about how they would try to create an appropriate low-tech, home-like environment, even in other more traditional maternity hospital settings:

*I feel like whenever I can actually get people to have babies at home or if we do go to the hospital have a home birth in the hospital*—*meaning we don*’*t use a lot of the technology in the room and we push it out of the way and we kind of keep to ourselves a little bit*—*we can have a home birth in the hospital and some women will end up with these magical amazing births on the floor of the bathroom at the hospital which is just as normal as the magical birth in the tub in the living room and it just happened in a different environment but it*’*s because we didn*’*t engage with all of the rest of the equipment and I find what I need to do in those situations is because I*’*m experienced now and people know me and I have a reputation*, *I can close the door and the nurse can come in when I want her to and I can ask her to come in*.

Seven participants talked about guarding the physical and social space for the woman. Some participants talked about protecting women from other hospital staff who might want to intervene, whereas other midwives talked about how they had built up important relationships and trust with hospital staff over time so that they did not interfere in women’s care. They also talked about protecting the woman’s privacy and helping her and her partner to manage the expectations of others and the pressures arising from social media:

*I feel that women and their partners do much better with privacy and intimacy during the birth process and that*, *my role is to sometimes protect that privacy and intimacy first of all by educating them that that might be really important and to talk about you know the effect both positive and negative about um*, *support during that time can be or even just letting people know hey*, *we*’*re in labour*, *the Facebook kind of thing but you know keep it quiet*, *keep it down*, *don*’*t fritter the energy away by drawing other people to it or drawing the expectation that something*’*s happening rather than just letting something evolve* … *I think guarding the space by keeping the space as calm and quiet and private as possible is key and giving people tools to do that during the prenatal time to deal with over eager family members or friends*.

A related theme highlighted in the data was the importance of the woman having support from a partner, friend or doula:

*Try to make sure she*’*s well supported so for example with our teen clients that*’*s not always*, *well with any clients*, *but specifically with our teen clients that*’*s often not the case. It*’*s often the case that they are not well supported. So they may or may not have the partner in the picture*, *they may have an estranged relationship with their parents. I would say more so than on the average other clients we have so for them we make sure that we get them doulas*, *we make sure they have doulas and we do a lot more intentional labour work with them*.

#### Careful watching and waiting

Participants referred to their role in quietly monitoring progress and taking small steps from time to time to address issues as they arise, in order to *nudge* things back to normal:

*I think the most important thing from my perspective is that my grounding in normal helps me recognize the abnormal* … *it*’*s like a line*, *the straight line of normal and if there*’*s a wavering off to one side or the other you just try a little tiny subtle intervention to nudge it back to normal* … *like if the baby is malpositioned you know*, *ideas and ways*, *and position changes*, *manoeuvres we can do to try to get the baby into a better position*, *things like that* … *so you know if she*’*s dehydrated*, *hydrate her. If she*’*s tired give her some food*, *you know*, *those kind of things* … *if it*’*s getting tachycardic and you hydrate mum and you cool her off*, *if she*’*s in the tub you cool her down*, *get her out of the tub*, *those little nudges and then quite often it normalizes*, *stabilizes and then you*’*re not creating a problem*.

Three midwives referred to limiting the number of vaginal examinations they conduct in labour, preferring to monitor other signs of progress in labour:

*And so part of that is I do think doing a vaginal exam in and of itself is an intervention that can slow things down for the mom* … *and now I really think about why am I doing this*? *What information am I going to get*? *Things like that and I do think that helps keep things normal because you*’*re doing less unnecessarily vaginal exams which is an intervention*.

One midwife referred to having a quiet presence:

*I try to keep a fairly quiet presence*, *try to work out what the woman and her partner*, *or partners*, *whoever*’*s around her*, *are being able to sort of do themselves* … *I think it*’*s probably better to let women go into themselves if they want to do that*, *so trying to support the woman in the kind of personality and needs that she has*, *and keeping that low*-*key presence with things like monitoring being a subtle as it can be*, *and I don*’*t really care for doing regular VEs so it*’*s more about clinical indications or their impression rather than it*’*s been 2 or 4 h since your last one so therefore you have another one*.

Another midwife spoke about the importance of a midwife *being really present* to achieve a normal outcome:

*You have to be present and I think that*’*s one of the things that keeps birth normal*, *we can go on and on about all the tools and I think those are valuable*, *I*’*m not saying they*’*re not*, *but I think that the message is you can*’*t do it from a distance so you can*’*t be at home while someone*’*s labouring*, *get up come in and do the birth*. [*If you don*’*t*] *You will have a higher section rate*, *so part of that is you need to be in attendance to keep the birth normal and some of it is just to have an opinion about the strip*, *some if it is literally where you feel like you*’*re standing guard*, *not against bad people but against keeping the space for the woman private and without a lot of stuff going on around her that*’*s going to distract her just being in her labour. So that*’*s part of our philosophy as a team that we tell our patients* … *You don*’*t leave a woman pushing ever so I think constant presence*, *I*’*ve come to believe that that*’*s really important*.

#### Helping the woman to cope with labour

Participants talked about the ways in which they help women to cope with labour. These included ensuring women attend prenatal classes, answering women’s questions candidly, being honest with women—*not sugar coating but not scaring*, talking with them about the range of pharmacological and non-pharmacological means to manage discomfort and pain in labour, and ensuring women have arranged good support (someone to be with them) for labour:

… *you know a lot of it is attitude like I say and going through the positive part of pain in labour and*, *and using your rest periods really effectively*, *not getting ahead of yourself*, *trying to just stay in one contraction at a time and I teach them that prior to labour but I also reinforce that a lot in labour and then comfort measures like water*, *we have great showers in our hospital*, *we don*’*t have tubs which is unfortunate but it is true the hot water never stops running so lots of my clients spend long times in the shower and different position changes and heat and ice and all of those things that we can use. I definitely like to use all of them*—*the ball*, *going up and down the stairs*.

Midwives also spoke about the need to consider the woman’s ability to cope and that sometimes it is too much to expect the woman to go without interventions:

*I always have held this in my heart*—*don*’*t sacrifice the relationship between the mother and baby because you want the woman to have a completely unintervened birth. I*’*ve seen that many times where a mom is so miserable by the time she has her baby and so exhausted and so out of it that she*’*s not even happy to see her baby when*, *you know as you become more and more experienced you realize there are ways that you can help that to be avoided*.

Two midwives talked about offering a woman an epidural if she gets to the point where it appears she *cannot do it anymore*, or where the woman has *crossed the line between pain and suffering*:

*But then the other situation is really straight up for pain relief when I*, *most of the time*, *when I would recommend an epidural is when I see*, *and am I wondering if that woman is crossing the line between pain and suffering. And so I*, *when I do offer that I could offer it very judicially and very gently but I would say something like* “*what I*’*m seeing here is this*, *I*’*m wondering if you might benefit from some pain relief*” *and basically either they jump on it or they*’*re like* “*no*, *no*, *I can manage*”.

#### Tools in the tool kit

The final cluster of themes related to different tools that midwives gather as they become more experienced and that they use in different scenarios to keep birth normal:

*It*’*s like tools in your toolkit and you*’*re filing that away and it*’*s like that idea of lifelong learning*, *you*’*re always going to be adding tools to your toolkit*, *you shouldn*’*t let it get full of cobwebs like you need to keep adding to it because there*’*s always something that*’*s going to work and make it a little change and for me the mechanics of it* … *and understanding all of those mechanics and bringing that to the mechanics of the pelvis and how babies come down and all of that and so there is a part of me that kind of*, *I can think it*’*s very cool that there are ways that this baby can come down and the more experience you get the more you realize yeah we can nudge this a little bit. Change that position*, *tilt*, *do this*, *do that*.

Midwives referred to a number of interventions that they used *wisely* to keep birth normal:

… *we can run into little obstacles on our way and  there are tools available and mostly they are my clinical skills but occasionally I suggest an epidural or maybe the patient really demands one and I have not enough to offer that she can do without*, *yeah of course*, *interventions need to be used wisely in order to achieve that goal* … *So I*’*m open to anything* … *I use a lot of alternative*, *I pretty much use any tool that is available*, *hopefully in the right situation to achieve that goal*.

Tools also included referring women for acupuncture and one midwife had basic training in acupuncture:

*I only have basic training in acupuncture so I sometimes do some*, *I sometimes send them to a practitioner* … *so I just do routine things like birth preparation if*, *induction points*, *turning the baby if it*’*s a breech baby so I just do basic things* … *we have some really good acupuncturist in town so I can send them out. I might use it in labour if her contractions slow down. If she*’*s not letting go and just I go again a little bit by my feelings and say okay this would benefit her in this situation*.

One midwife referred to approaches she used for cervical ripening for women with a history of post-term birth. Three other midwives referred to using labour cocktail or Verbena cocktail (a cocktail of castor oil and Verbena Officinalis) for the induction of labour:

*And a lot of that is about cervical ripping and stuff so with people who have a history of going postdates*, *I really encourage them to do stretch and sweeps and acupuncture and we do like cervical ripping tea and stuff like that. So we try to help them get ready at least to help their cervix ripen before we have to do something more serious and then if they don*’*t have a history*, *like I would say we do the same*, *basically the same thing. We just maybe leave it up to them a little more*.

Several midwives mentioned using water to help women to cope with pain and discomfort in labour:

… *we have a high water birth rate here*, *mostly because we have this water birth room*, *which is available with tons of hot water and a big beautiful tub and so essentially if I get them in the room*, *I get them in the water*, *I*’*m listening to the baby*, *I shut the curtains around the tub*, *I turn the lights down and I just give them that hour*, *like 1 to 2 h of kind of privacy where I*’*m sneaking in to listen to the baby*.

One midwife referred to rupturing membranes to rotate babies or to move the labour along. Other tools in the toolkit that midwives also referred to included ensuring the woman has adequate nutrition and hydration in labour, keeping the woman active, and homeopathy and hypnotherapy to help the woman to relax and to reduce anxiety.

## Discussion

The demand for midwifery care in BC is growing year on year. The promotion of normal birth is a key part of the midwifery philosophy of care and women who have their maternity care provided by a midwife are less likely to have a CS and other interventions. Recent research suggests care with a midwife is as safe as that provided by a family physician or obstetrician, regardless of whether the woman chooses to give birth with a midwife at home or in the hospital [17–19]. The findings in this study provide considerable insight into the strategies that midwives use to achieve this.

A key theme running throughout midwives’ accounts was continuity of care and the importance of the relationship between the woman and her midwife and of the midwife working with the woman from early in her pregnancy towards achieving a normal birth. In the BC model of midwifery provision, the emphasis is on the woman seeing the same care provider (midwife) for most of her care. A woman may also see mostly the same provider if her care is provided by a family doctor, but women attending for obstetrician-led hospital-based care may see different members of the multidisciplinary team providing that care. In BC in 2014/5, 51% of all women received their care from an obstetrician [20]. Key aspirations in continuity of care are woman-centred care, reducing the number of carers a woman sees, the midwife knowing the woman and the woman knowing the midwife, a woman being cared for by people who are familiar to her and aware of her plans for her birth, and her care provider providing a high degree of support in labour [27]. A systematic review of 15 trials [28] identified that women receiving care in midwifery-led continuity models of care were less likely to experience interventions and more likely to be satisfied with their care. Interestingly, the review found no impact on caesarean section rates. However, in the models of care that they examined all women gave birth in the hospital setting.

A key theme in the data was the importance of managing early labour. Early labour is a time of considerable uncertainty and it can be a very anxious time for women. Women may not know what to expect and they may need and seek reassurance about whether labour has started and when to attend the hospital [29]. Midwives in this study talked about the importance of preparing women for what to expect in early labour. They also talked about visiting women at home in early labour to assess them and to provide reassurance. A randomised control trial conducted in BC [30] found home visits in early labour to be more effective than telephone triage in reducing the number of women attending the hospital for assessment before they are in labour and those attending before 3 cm cervical dilation, although the home visits had no impact on CS rates or birth outcomes. What is different with midwives in this qualitative study is that the home visit in early labour was not a single intervention but one part of a comprehensive package of midwifery care in early labour. Midwives talked about a number of ways in which they help women to cope with early labour and ensure they do not get exhausted before they go into active labour, encouraging them to rest, mobilise, eat and drink. They reported recommending distraction, pharmaceutical and non-pharmaceutical pain relief and comfort measures, and Dimenhydrinate (Gravol^®^) to help women to rest. In BC midwives may administer Gravol^®^ under Midwives Regulations [31], for its sedative effects for “therapeutic rest during prodromal or early labour, particularly where anxiety is a factor” (p.40).

Midwives also reported encouraging women to stay at home for as long as possible in early labour in order to avoid the interventions associated with hospital. Cheyne and Hundley [32] suggest this is not surprising given the uncertainty that practitioners experience around deciding whether labour has started. They highlight that in the hospital the situation is fraught with anxiety, emotion, time pressures and competing priorities, and decisions are often based on unclear or incomplete information. It is acknowledged that hospitals tend to be optimised for high-risk women—with technology and staffing for close monitoring and quick access to interventions, and for low-risk women—staff monitor and tend to intervene more than is necessary [33]. This may result in avoidable harms to women and newborn while driving up the costs of maternity care. Miller and colleagues [34] use the term “too much, too soon” to refer to the over-medicalization of birth following the rapid increase in the use of hospital as the place of birth in high- and middle-income countries, which they claim, might offset recent gains from improvements in maternal and perinatal health. Their particular concern is the “trends towards excessive, unnecessary, or inappropriate use of obstetric interventions” (p.2178), including unnecessary ultrasound examinations, routine electronic fetal monitoring, routine episiotomy, high rates of labour induction and augmentation, and non-medically indicated CS.

The findings also emphasise the importance of women having a supportive birth environment and although midwives suggested that the optimal place of birth to facilitate normal birth is at home, they also described how they were able to adapt a hospital birthing space to facilitate normal birth and suggested that it is the responsibility of the midwife to protect the birth space, regardless of where the birth takes place. A qualitative study in Australia [35] highlights the medicalised environment in the hospital setting and its associated “biomedical discourse with an emphasis on risk” and how this can hinder the midwife’s ability to facilitate normal birth. There may also be a pressure to conform in this environment and difficulty challenging practice that is not evidence-based. Specific strategies that midwives used in that context to facilitate normal birth included “making a safe place for women and guarding the door” to exclude medical staff who may be inclined to take over at any time.

Fahy and Parratt [36] refer to the ideal birthing environment as the “sanctum”—a homely, private environment where the woman feels at ease and comfortable. The environment is familiar and feels private and safe, and has easy access to a bath, toilet and the outdoors. This environment supports “the woman’s embodied sense of self”, optimal physiological function and emotional wellbeing. They contrast this to the “surveillance space”—filled with equipment, dominated by the bed and lacking privacy, and suggest that the further the birth space is from the sanctum, the more likely the woman is to feel fear. Fourneur and colleagues [37] provide a comprehensive review of the research relating to the bio-behavioural system in labour to show how stress and fear in labour mediates the release of endogenous oxytocin, which can hinder the progress of labour, infant attachment and feeding. In another paper, Fourneur highlights the midwife’s role in guarding the birth space to ensure it is optimal for the woman and her baby. It is suggested that if the labour room has a technological theme, this emphasises birth as a biomedical event rather than a health event and can be a source of stress and fear for the woman. It is also suggested that the design of the birth unit and the model of care are important mediators in communication between a woman and her care providers and may also impact on staff’s stress and quality of decision-making [38].

The findings of this study also highlight that in the birth space the midwife needs to be “*really present*”. This further highlights the importance of the relationship that is developed between the woman and her midwife. A study of women’s negative experiences in labour [39] found the lack of presence (even though the midwife was in the room) and lack of feeling supported by the midwife, to be a central issue in women’s negative experiences. Midwives in this study highlighted the importance of women having good support in labour, both from a partner, family, and/or a doula and from the midwife. In the BC context, continuity of care from the midwife occurs across the full spectrum of care, including the birth. A systematic review of 22 studies [40] found women who had continuous support in labour (either from a nurse, midwife, doula, childbirth educator, family member, partner, or stranger) were more likely to have a spontaneous vaginal birth, a shorter birth, and to be satisfied with their birth.

Perhaps the most significant finding in this study is that midwives judiciously employ a spectrum of interventions to “nudge” labour and birth towards positive outcomes. This study highlights a number of interventions that midwives use to keep birth normal, dispelling the notion of normal labour and birth being free of interventions. Midwives clearly use interventions but these may not be regarded as medical or technological interventions. A study with exemplar midwives [41] describes midwives using themselves as “instruments of care” through their presence with the woman and using a finely tuned mix of low- and high-technological interventions to meet women’s needs. This mix, the authors suggest, is the difference between midwifery and medical models of care.

The strengths of this exploratory study lie in the insight generated from the detailed accounts provided by midwives. This is the first study on this topic to be conducted in BC and the findings support to-date anecdotal accounts of midwives’ commitment to promoting and supporting normal birth, some of the challenges they face, and identify a number of topics for further exploration. The limitations of the research include the small sample size overall, and that although the design aimed to allow for likely contextual and practice differences across BC, the very small numbers of midwives within each stratum further limits the applicability of the findings. In qualitative research, decisions about the generalizability or transferability of findings must be made by the reader, but this relies on the researcher providing adequate information on the methods and contexts used in the research [42]. The detailed examples provided in midwives’ accounts may usefully assist the reader in this regard, but further research is required to explore the topic more fully and to examine the impact of the interventions identified (e.g. management of early labour, home visits in early labour, delayed admission to hospital, water birth, Verbena cocktail) on the progress of labour and birth outcomes. Further research is also required to explore the experiences of other maternity care providers in BC (family doctors, obstetricians, and nurses).

## Conclusions

Midwives in BC have an important role in promoting and supporting normal birth as “instruments of care”. This begins early in pregnancy, working closely with women to prepare them for a normal birth, ongoing education, informed choice discussions and the management of expectations. Midwives use a range of midwifery skills and midwifery interventions across the course of care to “nudge” the pathway of pregnancy, labour and birth to the normal. The model of midwifery care, which emphasizes continuity of care, is a vital foundation for this.

## Additional file

Additional file 1: Semi-structured interview guide. (PDF 1329 kb)

## Abbreviations

BC: British Columbia
CS: Caesarean section
