# Supplementary material for: Exploring the strategies that midwives in British Columbia use to promote normal birth
Source: BMC Pregnancy Childbirth. 2017 Jun 5;17:168. doi: 10.1186/s12884-017-1323-7 (PMC5460538; doi:10.1186/s12884-017-1323-7)
Supplement: Additional file 1: — Semi-structured interview guide. (PDF 1329 kb) [file 12884_2017_1323_MOESM1_ESM.pdf]

a place of mind
THE UNIVERSITY OF BRITISH COLUMBIA

MIDWIFERY AT UBC

Exploring midwives' perceptions and experiences of normal birth

Interview Guide

Pre-interview briefing/ consent

i) Thank you for taking the time to talk with me. As I explained in my letter to you, this study aims to explore the perceptions and experiences of midwives of normal birth. The findings will be used to raise discussion and to inform practice. We will also use the findings to plan further research.
I also explained that I would like to record this interview with your permission, so that we have an accurate account of the discussion. We will then transcribe the recording in preparation for analysis. I can assure you that I will not reveal your identity to anyone and I will report all findings in a way that you cannot be identified.
ii) Are you still happy to take part in this interview?
iii) Would you like to ask any questions before we begin?

Obtain consent

1. To begin, could you tell me about your experience as a midwife in BC?
    a. How long registered
    b. Where you were before
    c. About your practice – e.g. solo, group, collaborative

2. What do you understand by the term normal birth?
    a. What does it mean in practice?
    b. How does it relate to natural birth?
    c. Does it include interventions?
        i. Epidural
        ii. Other forms of pain relief
        iii. Induction
        iv. Episiotomy
        v. Other interventions

3. What is your experience of normal birth in your daily practice as a midwife?
    a. How realistic is it?
    b. What is your own role in promoting normal pregnancy and birth?

4. Are their any particular challenges to maintaining normal birth?

5. What are the strategies that you use to maintain normal birth?

6. I have covered the questions I wanted to ask you, is there anything that you would like to add, or any questions that I should have asked?

Closing

Thank you for your time and participation. (Provide business card) Please contact me if you would like to discuss anything that we have discussed today further or if you have any queries or concerns about the study.
